# Repurposing Torrefied Biomass as a Novel Feedstock for Microbial Bioprocessing—A Proof-of-Concept of Low-Cost Biosurfactant Production

**DOI:** 10.3390/polym17131808

**Published:** 2025-06-29

**Authors:** Anjana Hari, Vahur Rooni, Udayakumar Veerabagu, Shiplu Sarker, Alar Konist, Timo Kikas

**Affiliations:** 1 Biosystems Engineering, Institute of Forestry and Engineering, Estonian University of Life Sciences, Kreutzwaldi 56, 51014 Tartu, Estonia; anjana.hari@emu.ee (A.H.); vahur.rooni@emu.ee (V.R.); udayakumar.veerabagu@emu.ee (U.V.); 2 Department of Manufacturing and Civil Engineering, Faculty of Engineering, Norwegian University of Science and Technology, 2815 Gjøvik, Norway; shiplu.sarker@ntnu.no; 3 Department of Energy Technology, Tallinn University of Technology, 19086 Tallinn, Estonia; alar.konist@taltech.ee

**Keywords:** torrefaction, thermochemical pretreatment, lignocellulosic waste, waste-to-wealth, biorefineries

## Abstract

Torrefaction is a thermochemical pretreatment in which biomass is heated at 200–300 °C for 30–60 min in an inert atmosphere. Torrefaction has been previously used to improve the fuel properties of lignocellulosic biomass; however, the use of torrefaction for bioenergy generation represents a low-value final product as well as the dead end of the biomass value chain. Herein, we demonstrate the proof-of-concept for the utilisation of torrefaction as a pretreatment to convert low-value wood waste into biosurfactants, a high-value specialty biochemical. Wood waste was torrefied at 225 °C, 250 °C, 275 °C, and 300 °C and physicochemically characterised using proximate and ultimate analyses, FTIR, XRD, TGA–DTG, and SEM–EDX to assess its suitability as fermentation feedstock. Aspen waste torrefied at temperatures less than 250 °C was directly utilised by *Burkholderia thailandensis* DSM 13276 via semi-solid-state fermentation to yield biosurfactants, and 225 °C was selected for further experiments as it resulted in the production of biosurfactants which reduced the surface tension of the production medium to 36.8 mN/m and had an emulsification index of 64.1%. Tension and emulsification activities decreased with the increase in torrefaction temperature. The biosurfactant derived from torrefaction at 225 °C formed highly stable emulsions with diesel oil (lasting >40 days), in addition to low interfacial tension, suggesting potential applications in diesel bioremediation. This integrated, chemical-free strategy offers an alternative application for torrefied wood waste as well as a feasible solution for the cost-effective chemical-free production of biosurfactants, incorporating circular economy principles.

## 1. Introduction

Wood is a renewable, biodegradable natural resource with mechanical and chemical properties that make it an important part of construction and manufacturing worldwide. Its newer applications include composite materials and even chemicals. Wood and wood products are crucial components of the Estonian economy, giving rise to a thriving timber industry that accounts for approximately 23% of total industrial manufacturing turnover, according to the latest available data [1]. However, the increase in consumption of wood has also resulted in the generation of considerable amounts of wood waste in the form of offcuts, shavings, and wood chips during the different processes of the wood industry value chain. Further, low-quality wood, such as damaged, rotten, crooked, and small-diameter logs, which cannot be used in furniture or the construction industry, are also generated [2]. Current practices of wood waste management involve landfilling, incineration for energy recovery, and material recovery [3]. In Estonia, most of these secondary woody biomasses or residues are used for district heating due to their high calorific value, as there are no alternative applications [4]. This also contributes to greenhouse gas emissions, causing environmental pollution. Thus, finding high-value applications for these wood wastes could offer solutions for waste management as well as pollution prevention. A potential solution is to use such minimally processed wood waste as a feedstock for potential biorefinery applications.

Wood is composed of three major structural components—cellulose, hemicellulose, and lignin, with their relative composition varying based on species [5]. Considering the diverse origin, types, and composition of these wood wastes, it is essential to perform a pretreatment that breaks down the complex structure of lignocellulose as well as confers homogeneity to it. Torrefaction is such a thermochemical pretreatment process, wherein biomass is heated at a temperature of 200–300 °C for 30–60 min in an oxygen-deficient environment at atmospheric pressure. Conventionally, torrefaction has been used to improve the fuel characteristics of biomass [6]. However, in terms of circularity and sustainability, using torrefied biomass as a fuel results in a dead-end, linear value chain. Moreover, torrefied biomass as a low-grade fuel has low value in terms of cost of the final product, limiting the business prospects of this pretreatment. Therefore, the integration of torrefaction with other biomass conversion processes could improve the relevance of this pretreatment in today’s bio-based production processes [7]. Torrefaction is a milder physicochemical pretreatment as it requires less energy input due to its lower operating temperature, shorter residence time, and ambient pressure requirement, which is an added benefit in reducing the raw material costs of bio-based production. Further, the volatile organic components released in gaseous form during torrefaction can be used to generate the process heat required to sustain the process, improving the economics further. Recently, the use of torrefaction as a pretreatment for fermentation has been reported via hydrolysis [6,8,9,10]. Torrefaction followed by enzymatic hydrolysis at lower temperatures yields monomeric sugars, which have been used as a platform for bio-ethanol production [11]. This led us to hypothesise that this pretreatment can be used as a strategy to produce more high-value compounds or specialty biochemicals such as biosurfactants. At present, the major product derived from torrefaction is torrefied biomass or bio-coal, which, according to a study, had the lowest selling price of 103–105 USD/ton [12]. Depending on the feedstock, the side-streams of torrefaction could include platform chemicals such as furfural and 5-(hydroxymethyl)furfural, which has a revenue per ton of USD 15 and 3, respectively [13]. Moreover, these are produced at very low yields via torrefaction, which justifies the search for high-value products [14].

Biosurfactants are surface-active compounds with hydrophilic heads and hydrophobic tails that reduce interfacial and surface tension. These versatile compounds are used in the medical, pharmaceutical, food, and petroleum industries, agriculture, bioremediation, and in cleaning and personal care product formulations [15]. They are preferable to chemical surfactants as they are biodegradable and less toxic [16]. Further, consumers are willing to pay a higher price for safer products with a lower environmental burden [17]. However, the commercial production of biosurfactants is not price-competitive in bulk applications at present due to the high cost associated with the procurement of raw materials and purification in addition to low yield [18]. For example, the minimum selling price of commodity petrochemical-derived surfactants such as LABSA is 1.3–2 USD/kg, whereas that of biosurfactants ranges from approximately 12 to 56 USD/kg [19]. The cost factor restricts their use to high-value niches such as medicines, medical coatings, and expensive cosmetics, which require a purity grade of ≥90%. Thus, it is beneficial to develop a feasible production strategy that combines a cost-effective substrate and focused microbe-mediated fermentation so that biosurfactants’ application may be extended in an economically feasible manner to other fields.

Herein, we aim to develop a proof-of-concept to produce biosurfactants using the underexploited torrefied biomass as a substrate and the bacterium *Burkholderia thailandensis* E264. The selection of this bacterium as the biosurfactant producer was based on two key reasons—(i) it has demonstrated biosurfactant production capability (rhamnolipids) and no known pathogenic activity, making it a safer choice for future industrial-scale processes [20], and (ii) *Burkholderia* spp. has been known to utilise cellulose, hemicellulose [21], as well as different aromatic compounds including lignin-derived compounds such as benzoic acid, vanillin, syringic acid, and syringol as sole carbon sources [22,23,24]. This strategy will significantly improve the overall economy of the torrefaction process by utilizing polysaccharide and lignin fractions to produce high-value-added products.

## 2. Materials and Methods

### 2.1. Biomass

Aspen (*Populus tremula*) wood chips were kindly provided by AS Estonian Cell located in Lääne-Virumaa, Estonia. Aspen is a commonly used hardwood in the Estonian timber industry. The wood chips were air-dried for two weeks indoors and oven-dried at 105 °C overnight before torrefaction to ensure that the initial properties would be as similar as possible.

### 2.2. Torrefaction Experiments

**Figure 1:**
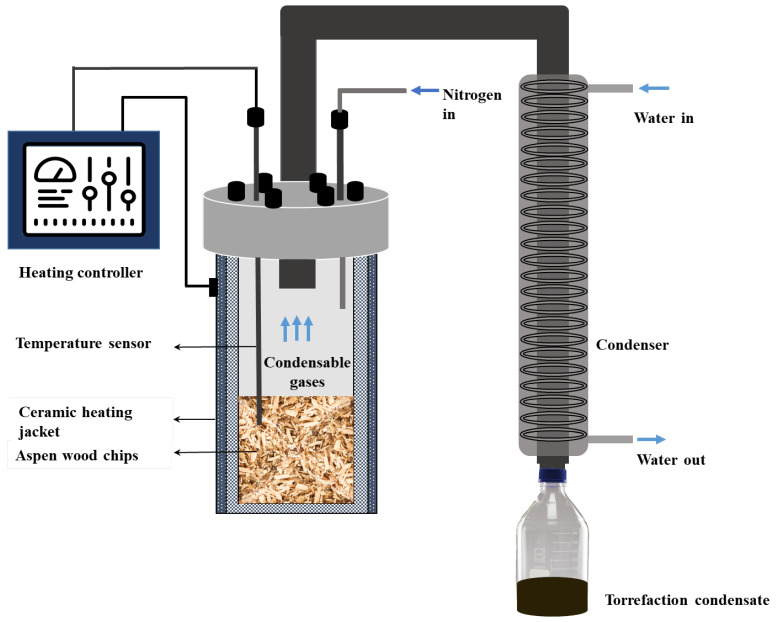
Batch-type torrefaction reactor.

A batch-type reactor system (Figure 1) was used for performing torrefaction. Approximately 150 g of oven-dried wood chips were weighed and transferred to the reaction vessel. The reactor assembly was completed by tightening the lid and attaching the condenser set-up. To maintain an inert environment, nitrogen gas was used, with a flow meter controller (Vögtlin Instruments GmbH, Herne, Germany). To ensure an inert atmosphere, prior to heating, the reactor was flushed with nitrogen at a rate of 8 L/min for 10 min, which was later reduced to 4 L/min. The reactor was heated using a ceramic band heater and controlled by a proportional–integral–derivative controller. Temperature data was constantly monitored using PicoLog data logger(version TC-08, Pico Technologies, Cambridgeshire, UK). Torrefaction was performed at four different temperatures (225 °C, 250 °C, 275 °C, and 300 °C) at a fixed residence time of 1 h. All condensable gases were collected via a cold-water condenser unit as a dark, aromatic liquid condensate. After torrefaction, the reactor set-up was allowed to cool, and the torrefied biomass was collected, weighed, and stored in air-tight glass containers for further analyses. Before further experiments, the torrefied wood chips were milled and sieved to a particle size of <4 mm using a Retsch SM100 mill (Retsch GmbH, Haan, Germany).

### 2.3. Characterisation of Torrefied Biomass

#### 2.3.1. Compositional Analysis

Different aspects of the physical and chemical composition of torrefied biomass were studied. The moisture content of the biomass before and after torrefaction was measured using a Kern MLS-50-3D moisture analyser (Kern & Sohn GmbH, Balingen, Germany). Approximately 30 mg of each torrefied biomass sample and untreated aspen samples were used for analysing the elemental composition [carbon (C), hydrogen (H), nitrogen (N), and sulphur (S)] using Elementar Vario Macro Cube analyser (Elementar Analysensysteme GmbH, Langenselbold, Germany). Oxygen content (O) was calculated as a difference using the following formula:

(1) O=100−(C+H+N+S)

Plant fibre content (cellulose, hemicellulose, lignin, and extractives) of the torrefied and untreated biomass was analysed using an Ankom 2000 Fiber Analyzer (ANKOM Technology, New York, NY, USA) according to the detailed protocol outlined in Cahyanti et al. [6].

Ash content was determined according to ISO 18122:2015 [25] protocol by gradually combusting 1 g of each different torrefied and untreated biomass in pre-weighed crucibles at 550 °C over a period of 4 h in a muffle furnace (Milestone PYRO Advanced microwave muffle furnace, Milestone™ Srl, Bergamo, Italy) and weighing the residue after cooling.

#### 2.3.2. Thermogravimetric and Differential Thermogravimetric (TGA–DTG) Analyses

The thermal degradation behaviours of the torrefied and raw biomass were studied from 200 °C to 800 °C in an N_2_ atmosphere using a thermal analyser (Netzsch STA 449 F3 Jupiter Simultaneous Thermal Analyser, Selb, Germany).

#### 2.3.3. Fourier Transform Infrared Spectroscopy (FTIR)

FTIR (Spectrum BXII, Perkin Elmer Inc., Hopkinton, MA, USA) was used to study the surface functionalities of raw and torrefied biomass using the universal attenuated total reflection (ATR) method. The spectra included 4000–600 cm^−1^ interval range at a resolution of 4 cm^−1^, and an accumulation of 20 scans.

#### 2.3.4. X-Ray Diffraction (XRD) Analyses

The crystallinity of the raw and torrefied biomass was determined by XRD (X’pert Pro Panalytical, Almelo, The Netherlands) and the data were recorded over a diffraction peak position (2θ) range of 5–90° using Cu Ka radiation at 40 kV and 40 mA. The crystalline indices (*I_c_*) of thermally treated aspen samples at various temperatures were calculated using the following formula.

(2) $$I_{c}\left(\%\right)=\frac{A_{c}}{A_{c}+A_{a}}\times 100$$

where *A_c_* is the area of all crystalline peaks and *A_a_* is the area of all amorphous peaks.

#### 2.3.5. Scanning Electron Microscopy and Energy Dispersive X-Ray Analysis (SEM-EDX)

The surface morphology of the torrefied biomass was analysed using SEM equipped with energy dispersive X-ray (EDX) (JSM 6390LV, JEOL Inc., Tokyo, Japan).

### 2.4. Biosurfactant Production Using Torrefied Biomass as Carbon Source

#### 2.4.1. Microbial Culture and Media

Freeze-dried culture of *Burkholderia thailandensis* (DSM 13276) was purchased from Leibniz Institute DSMZ-German Collection of Microorganisms and Cell Cultures GmbH, Braunschweig, Germany. The culture was revived in nutrient broth [peptone (VWR International GmbH, Darmstadt, Germany) 5.0 g; beef extract 3.0 g, and distilled water 1 L; pH 7] according to DSMZ protocol and stored as 30% (*v*/*v*) glycerol (Fisher BioReagents, Pittsburgh, PA, USA) stocks at −80 °C till use. Before biosurfactant production, a loopful of culture from a glycerol stock was used to inoculate a nutrient agar [same as nutrient broth, supplemented with 1.5% (*w*/*v*) agar (Thermo Fisher Scientific, Waltham, MA, USA)] plate, which was incubated at 28 °C. Single colonies were used to make a starter culture, incubated overnight at 28 °C with shaking at 220 rpm.

To determine if torrefied biomass could be directly used as a substrate for biosurfactant production, 5% (*w*/*v*) of each torrefied biomass sample was added to 200 mL of nutrient broth and autoclaved at 121 °C, and 15 psi of pressure for 15 min. Nutrient broth supplemented with 4% (*v*/*v*) glycerol was used as a control for biosurfactant production. An inoculum size of 2% (*v*/*v*) was used for each experiment. The production media were incubated at 28 °C with shaking at 220 rpm till 192 h, and samples were taken periodically to measure biosurfactant production.

#### 2.4.2. Cell Separation and Evaluation of Tensio-Activity, Emulsification, and Biosurfactant Production

The production media were harvested after cultivation, and the microbial cells and residual biomass were separated by centrifugation at 12,000 rpm for 15 min. The cell-free supernatants were stored at 4 °C and analysed for surface tension reduction, emulsification, and biosurfactant concentration.

Surface tension was analysed using a Sigma 701 (Biolin Scientific, Gothenburg, Sweden) tensiometer, using a platinum Du Noüy ring. The tensiometer was calibrated by measuring the surface tension of methanol before each sample measurement.

To assess the ability of the produced biosurfactants to form and maintain emulsions, 3 mL of each cell-free supernatant and 3 mL of diesel oil were vortexed together in a clear glass vial for 2 min (or till emulsion formation for higher torrefaction temperatures) and allowed to settle. A 1% (*w*/*v*) solution of cetyltrimethylammoniumbromide (CTAB; VWR International GmbH, Darmstadt, Germany) was used as the positive control. Emulsification indices were calculated after 24 h, using the following formula:

(3) $$E_{24}(\%)=\frac{H_{E}}{H_{T}}\times 100$$

where *E_24_* is the emulsification index calculated after 24 h, *H_E_* is the height of the emulsion layer, and *H_T_* is the total height of the liquid layers.

The stability of the emulsions was studied by leaving the tubes at room temperature for up to 2 months. Stable emulsion layers were extracted using a sterile microtip and examined under a phase contrast microscope under 10× and 40× magnification (Zeiss Primostar 3, Carl Zeiss Microscopy GmbH, Jena, Germany).

Biosurfactant concentrations in each of the cell-free supernatants derived from different torrefied biomass were measured indirectly as a function of total sugar content using the orcinol method. To 100 µL of each cell-free supernatant, 900 µL of freshly prepared orcinol reagent [0.19% (*w*/*v*) orcinol (Sigma-Aldrich, St. Louis, MO, USA) in 53% (*v*/*v*) H_2_SO_4_ (Merck Chemicals, Darmstadt, Germany)] was added. After incubating at 80 °C for 30 min in a heating block, samples were cooled to room temperature, and the absorbance was measured at 421 nm with a UV–visible spectrophotometer (Macherey-Nagel Nanocolor UV/VIS II, Düren, Germany). Standard curves were prepared using L-rhamnose (Sigma-Aldrich, St. Louis, MO, USA), and the concentration of rhamnose in each cell-free supernatant was calculated from standard curves prepared in the range of 0–5 mg/mL.

Those cell-free supernatants demonstrating good tension-active and emulsifying properties were subjected to other qualitative assays for biosurfactant production, to determine properties such as net charge and hemolytic activity, and identify possible applications.

#### 2.4.3. Qualitative Assays for Biosurfactant Production

##### Hemolysis Assay

Blood agar plates were prepared by adding 5% (*v*/*v*) de-fibrinated sheep blood (Sigma-Aldrich, St. Louis, MO, USA) to blood agar base (VWR International GmbH, Darmstadt, Germany). Wells of 6 mm size were made in the plate and 150 μL of cell-free supernatant was added to the wells and incubated at 28 °C overnight [26]. Cell-free supernatant from *B. thailandensis* grown on nutrient broth with 4% (*v*/*v*) glycerol was used as the positive control.

##### CTAB-Agar Assay

CTAB-methylene blue agar plates were prepared by adding 0.78 g of CTAB, 0.002 g of methylene blue (Thermo Fisher Scientific, Waltham, MA, USA), and 1.5 g of agar to 1 L of distilled water (pH 7). Two wells of 6 mm size were made in the plates and 150 μL of cell-free supernatant was added to the wells and incubated at 28 °C overnight [27]. Commercial rhamnolipids (AGAE Technologies LLC, Corvallis, OR, USA) were used as the positive control. De-ionised water was used as a negative control.

##### Drop-Collapse Assay

To screen for biosurfactant production in media containing torrefied biomass, drop-collapse tests using crude oil [28] were performed. Here, 5 μL of respective oils were placed to the well demarcations on the lids of a polystyrene 96-well microplate and allowed to equilibrate for 24 h at room temperature. Approximately 2 μL of cell-free culture supernatant was transferred to the coated areas and the drop was observed. De-ionised water was used as a negative control. Cell-free supernatant from *B. thailandensis* grown on nutrient broth with 4% (*v*/*v*) glycerol was used as the positive control. The results were recorded within 10 min.

##### Oil Displacement Assay

To a plastic Petri dish containing 15 mL of de-ionised water, 40 μL of crude oil was added [29]. Then, 10 μL of cell-free supernatant was added to the surface. De-ionised water was used as a negative control. Cell-free supernatant from *B. thailandensis* grown on nutrient broth with 4% (*v*/*v*) glycerol was used as the positive control. The results were recorded immediately.

##### Analysis of Interfacial Tension

A spinning drop video tensiometer SVT20N (DataPhysics Instruments, Filderstadt, Germany) was used to visualise and measure the ability of the cell-free supernatant to reduce the interfacial tension of crude oil and diesel. A clean fast exchange capillary (FEC 622/400) was partially filled with the denser phase (cell-free supernatant) using a glass Pasteur pipette. Using a sterile 80 mm syringe, a single drop of the lighter phase (crude oil or diesel) was carefully injected into the capillary, and it was closed after filling the rest of the capillary with the cell-free supernatant. After fitting the capillary into the tensiometer, measurements were made using the associated software (version SVTS 20) of the spinning drop tensiometer. The positive control used was cell-free supernatant from *B. thailandensis* grown on nutrient broth with 4% (*v*/*v*) glycerol. At least two measurements of dynamic interfacial tension were taken for each sample, and the results were expressed as mean of the two measurements. The value was considered valid if the camera error was <0.5.

### 2.5. Statistical Analyses

Unless otherwise mentioned, all the experiments were conducted in triplicate. The results are expressed as mean ± standard deviation.

## 3. Results and Discussion

In this work, we comprehensively characterised torrefied biomass and determined the conditions of torrefaction that allow the utilisation of torrefied biomass as a potential feedstock for the production of high-value specialty chemicals such as biosurfactants. Our findings are described below.

### 3.1. Chemical and Physical Characterisation of Torrefied Biomass

#### 3.1.1. Elemental Composition of Raw and Torrefied Aspen Biomass

The elemental compositions of the raw and torrefied biomass were analysed to evaluate the impact of torrefaction-induced changes on the chemical composition of the waste aspen biomass. As depicted in Table 1, the increase in torrefaction severity from 225 °C to 300 °C led to a gradual increase in the carbon content, which was also accompanied by a reduction in the oxygen and hydrogen concentrations. The raw aspen biomass exhibited a carbon content of 47.7%, which steadily increased to 61.4% (300 °C). Conversely, the oxygen content decreased from 45.0% in the raw aspen to 32.6% at increased torrefaction temperatures. In addition, the hydrogen content of the torrefied biomass decreased from 7.1% to 5.9% at 225–300 °C. These compositional shifts were reflected in the corresponding van Krevelen molar ratios. The atomic O/C ratio decreased significantly between 0.94 (raw aspen) and 0.53 (300 °C), which is attributed to the substantial deoxygenation due to devolatilisation and the breakdown of hemicellulose and amorphous cellulose in biomass during torrefaction [30,31]. Similarly, the H/C ratio also reduced from 0.15 to 0.096, reflecting progressive dehydration possibly related to the rapid cleavage of hydroxyl groups as the temperature increased [32]. These observations align well with the previous reports about the enhanced carbonisation of biomass with increasing torrefaction temperature [32,33,34]. Sulphur was negligible in the biomass samples.

#### 3.1.2. Fibre Analysis of Raw and Torrefied Aspen Biomass

**Figure 2:**
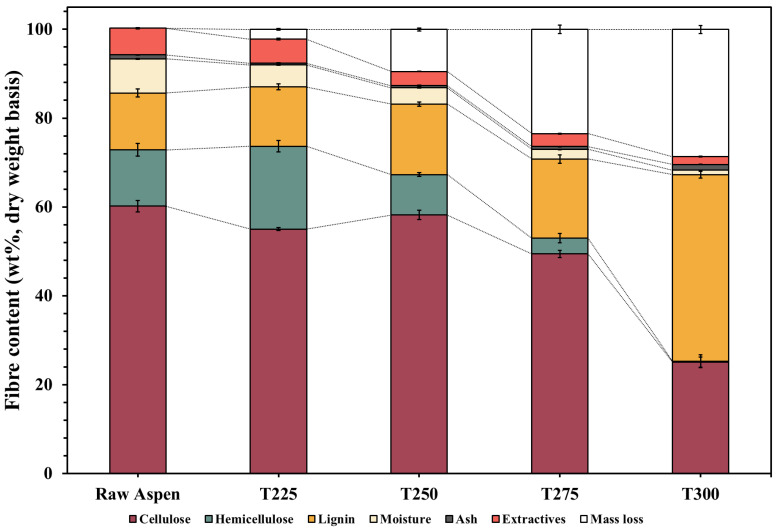
Fibre composition of untreated and torrefied wood waste.

The effect of torrefaction on the compositional analysis of the raw and torrefied aspen biomass illustrated a distinct transformation in the fibre constituents with increasing temperature (Figure 2). In the raw aspen biomass, cellulose constituted 60.2%, followed by hemicellulose (12.7%), and lignin (12.8%). Upon torrefaction at 225 °C, a slight reduction in cellulose (55%) was observed, while the relative hemicellulose content unexpectedly increased to 18.7%. It is well-established in the literature that the hemicellulose content degrades first during torrefaction. We speculate that the increase in our study is most likely because of the chemical modification (opening up) of the hemicellulose structure and the attachment of other components [extractive compounds derived from hemicellulose breakdown (which cannot be measured by the instrumentation used in the study) or glucose moieties from partial breakdown of amorphous cellulose] to the open structure. This could probably occur if other components (like extractives) degrade even more rapidly than hemicellulose [35]. These hemicellulose-like materials, which are partially degraded, become more detectable during analysis. Moreover, the reduction in volatiles, moisture, and extractives may increase the relative concentration of hemicellulosic fibres in the composition of pretreated biomass [36]. A study on torrefaction of pine logging residues also reports a slight increase in hemicellulose after torrefaction at 225 °C [37]. As the torrefaction temperature further increased, both cellulose and hemicellulose showed significant degradation. At 300 °C, the cellulose content decreased sharply to 25.1%, whereas hemicellulose was nearly depleted (0.2%). This observation validates the thermolabile nature of cellulose and hemicellulose, with a significant breakdown of their polymeric structures beginning above 240 °C [38]. In contrast, the lignin content increased from 12.8% in the raw aspen to 42% at 300 °C. This gradual increase was attributed to the relative resistance of lignin to thermal decomposition [39] and the potential formation of pseudo-lignin from the degradation by-products of cellulose and hemicellulose [11]. Pseudo-lignin is believed to be an inhibitor compound containing carbonyl groups, formed from the degradation of the hemicellulose component due to dehydration reactions under milder torrefaction conditions and from the cellulose component after severe torrefaction in the range of 300 °C. Furfurals/pseudo-lignins (aldehyde) are reported to inhibit hydrolysing enzymes via unproductive binding [40]. The moisture and extractive contents decreased with increasing temperature, indicating the dehydration of the polymer matrix and volatilisation of low-molecular-weight compounds, respectively [31,32]. In parallel, the ash content increased from 1% in raw aspen to 1.4% at 300 °C because of the concentration of mineral residues following the release of volatiles [41]. The overall mass loss increased from 2.2% at 225 °C to 28.6% at 300 °C, highlighting the increasing levels of devolatilisation and structural breakdown [42]. These findings are consistent with the well-documented degradation sequence of lignocellulosic biomass in the literature, where hemicellulose decomposes at lower temperatures, followed by cellulose, while lignin remains relatively recalcitrant.

#### 3.1.3. TGA–DTG Analyses

**Figure 3:**
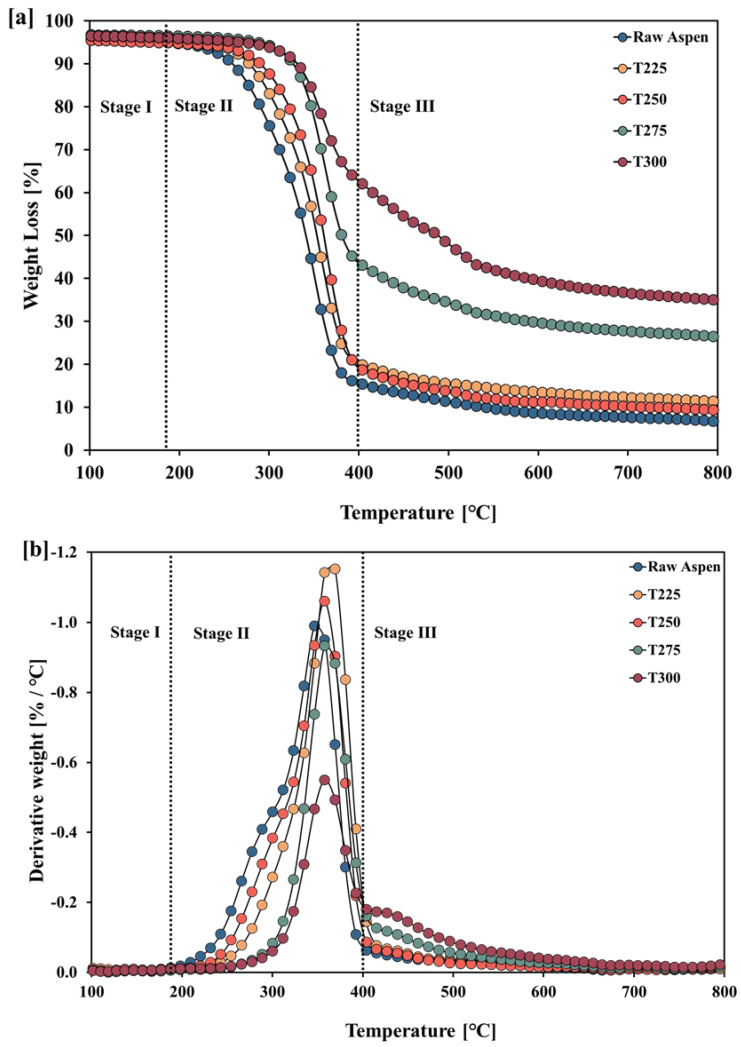
TGA–DTG analyses of untreated and torrefied wood waste showing different stages of thermal degradation.

TGA and DTG analyses for raw aspen and torrefied aspen at different temperatures are shown in Figure 3a and Figure 3b, respectively. The samples were heated from 25 to 800 °C at a constant heating rate of 20 °C/min under an N_2_ atmosphere. The thermal degradation of lignocellulosic biomass components typically occurs within the ranges of 200–315 °C for hemicellulose, 315–400 °C for cellulose, and 250–500 °C for lignin [33,34,43]. In Stage I, meagre weight loss due to moisture evaporation was observed in both untreated and torrefied aspen, primarily because both raw and torrefied materials were dried at 105 °C prior to analysis [43]. Stage II corresponds to the primary decomposition phase. During this phase, in the DTG curve, a shoulder peak appears only for the raw aspen sample, while broader, merged peaks are obtained for 250 °C and 225 °C due to the partial degradation of hemicellulose followed by the formation of more stable hemicellulose residues in this temperature range, which then decompose simultaneously with cellulose [44]. A significant weight loss of approximately 70% was observed between 220 °C and 400 °C, as indicated by TGA and DTG curves. This is attributed to the complete breakdown of hemicellulose and cellulose. During Stage III, an additional weight loss of around 25% was recorded between 400 °C and 600 °C, represented by a broad region in the TGA curve and a small DTG peak, reflecting the gradual degradation of lignin. Above 600 °C, no significant weight loss was detected, indicating the formation of char and the completion of biomass conversion.

#### 3.1.4. FTIR Analysis

**Figure 4:**
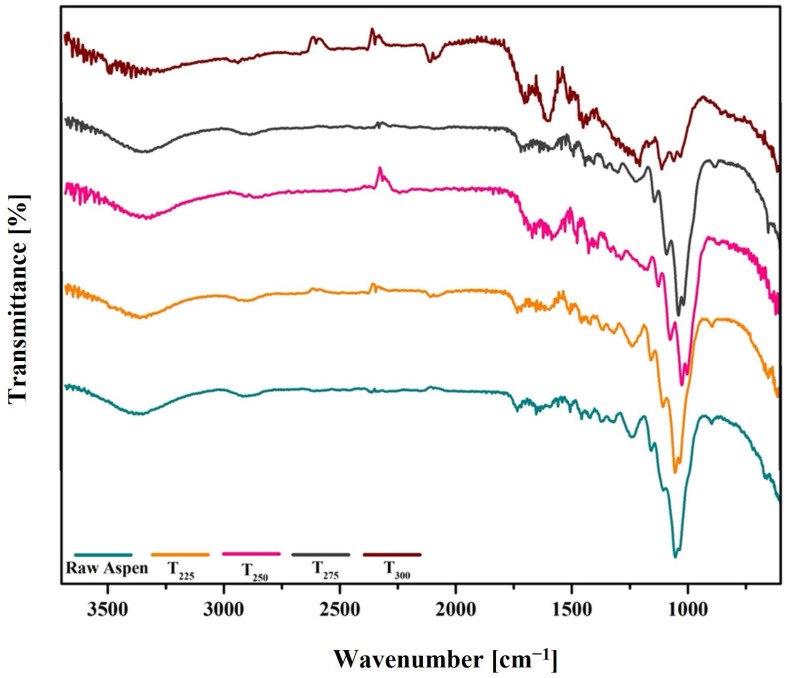
FTIR spectra of untreated and torrefied wood waste.

The FTIR spectra of raw and torrefied aspen samples at various temperatures are shown in Figure 4. The broad absorption band between 3600 and 3200 cm⁻^1^ corresponds to O–H stretching vibrations due to moisture and hydroxyl groups present in cellulose, hemicellulose, and lignin [45]. The intensity of this absorption band decreased notably with increasing torrefaction temperature, indicating moisture loss and hydroxyl group decomposition. C–H stretching vibrations associated with polysaccharides and lignin appeared between 3000 and 2800 cm⁻^1^, and their intensity diminished progressively with higher pretreatment temperatures, demonstrating the breakdown and fragmentation of aliphatic C–H bonds [46]. Absorption peaks observed in the 1750–1600 cm⁻^1^ region correspond to C=O stretching vibrations from carbonyl groups in lignin, esters, acetyl groups in xylan, and carboxylic acids [46,47]. The intensity of these peaks decreased significantly in the torrefied samples, reflecting hemicellulose decomposition and loss of associated oxygenated functional groups. The peak around 1510 cm⁻^1^ is assigned to C=C stretching vibrations within aromatic structures [43]. Additionally, distinct bands between 1250 and 1000 cm⁻^1^ are associated with C–O–C vibrations, C–H bending, asymmetric C–O–C stretching vibrations in polysaccharides, and C–O vibrations from guaiacyl rings and alcohols [47]. These bands were prominent in raw aspen but exhibited notable intensity reductions with increasing torrefaction temperatures, indicating substantial carbohydrate degradation. The FTIR results thus highlight progressive structural changes in biomass, characterised by moisture reduction, hemicellulose degradation, and volatilisation, ultimately forming a structurally condensed, carbon-rich material [33].

#### 3.1.5. XRD Analysis

**Figure 5:**
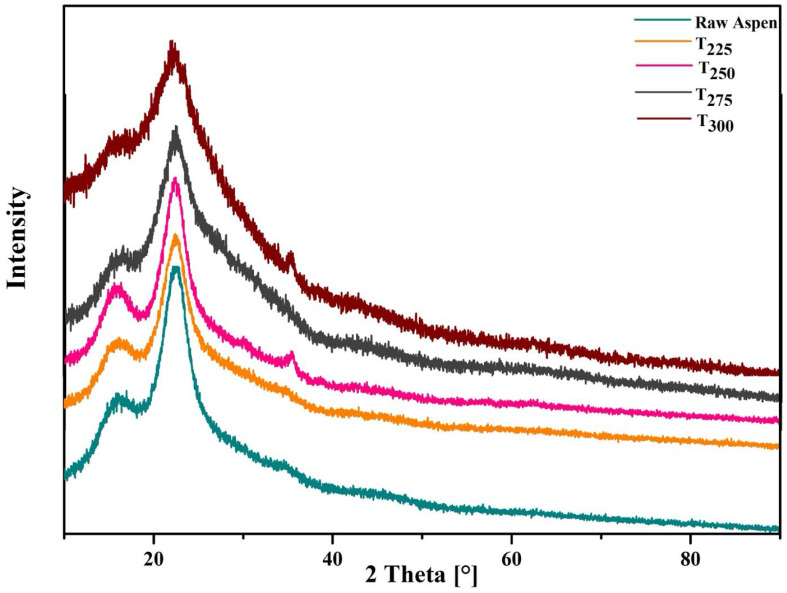
XRD patterns of untreated and torrefied wood waste.

The XRD patterns of raw aspen and torrefied aspen at different temperatures are presented in Figure 5. For raw aspen, sharp crystalline peaks at 2θ values of 16.2° and 22.5° were observed, corresponding to the cellulose crystalline planes (101) and (002), respectively. Thermal pretreatment at 225 °C and 250 °C did not significantly affect the crystalline nature of cellulose, as indicated by the persistence of sharp peaks at these positions. However, upon further pretreatment at higher temperatures (275 °C and 300 °C), a noticeable broadening and diffuse scattering of these peaks occurred, suggesting the breakdown of cellulose chains as well as potential lignin condensation [43]. Raw aspen waste showed the highest crystalline index of 75%. For thermally treated samples, I_c_ values of 72% and 70% were noted at 225 °C and 250 °C, respectively, indicating only a slight impact on crystallinity at these temperatures. In contrast, substantial decreases in I_c_ to 48% and 40% were recorded at temperatures of 275 °C and 300 °C, respectively. These reductions highlight a significant breakdown of cellulose crystallinity at elevated temperatures [48].

#### 3.1.6. SEM-EDX Analysis

**Figure 6:**
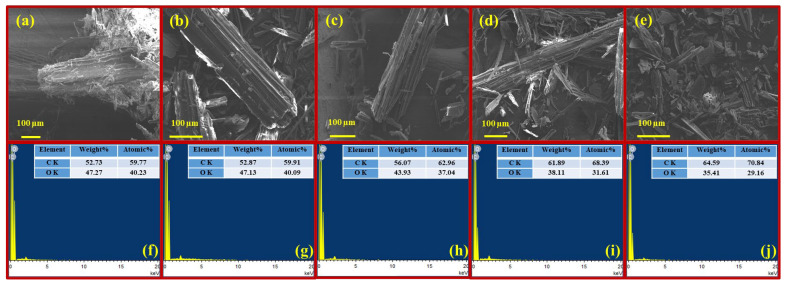
SEM images of untreated and torrefied wood waste: (**a**) Untreated biomass, (**b**) wood waste torrefied at 225 °C, (**c**) wood waste torrefied at 250 °C, (**d**) wood waste torrefied at 275 °C, (**e**) wood waste torrefied at 300 °C and EDX spectrogram of untreated and torrefied wood waste. (**f**) Untreated biomass, (**g**) wood waste torrefied at 225 °C, (**h**) wood waste torrefied at 250 °C, (**i**) wood waste torrefied at 275 °C, (**j**) wood waste torrefied at 300 °C.

The impact of torrefaction pretreatment on the structural integrity and elemental composition of the raw and torrefied aspen biomass was investigated using SEM–EDX analysis. As observed in the SEM images (Figure 6a–e), a clear transformation in surface morphology and increased structural deformation is detected with the increase in torrefaction temperature. Compared to the torrefied biomass, the raw aspen biomass displayed a smooth and intact fibrous structure with minimal surface deformation, which indicates the presence of hemicellulose and unaltered cellular arrangements. However, the increase in torrefaction temperature led to a decomposition of structural polysaccharides, resulting in the noticeable surface modification [49]. Mild torrefaction at 225 °C and 250 °C (Figure 6b–c) resulted in the increase in surface roughness, which also exhibited developments of cracks and voids, representing the onset of hemicellulose degradation and initial signs of biomass degradation [33]. As the torrefaction temperature reached 275 °C (Figure 6d), the biomass underwent more extensive deformation and fragmentation of the fibrillar matrix. These modifications suggest the partial volatilisation and breakdown of hemicellulose and the amorphous region of cellulose in biomass [50]. At the highest torrefaction condition of 300 °C (Figure 6e), extensive structural alteration occurred, characterised by a sharp increase in the presence of irregular fractured particles. This significant deformation is consistent with advanced thermal degradation, where lignin softening and volatilisation of low-molecular weight compounds at higher temperature contribute to biomass shrinkage and the development of ruptures [43,51]. The EDX spectra reveal that carbon (C) and (O) are the predominant elements present in both untreated and torrefied aspen biomass (Figure 6f–j). With increasing the torrefaction temperature, a consistent rise in carbon (52.87–64.59%) and decrease in oxygen (47.13–35.41%) content were observed. The shift in the elemental composition suggests the removal of oxygen-rich functional moieties through various thermal reactions (dehydration, decarboxylation, and decarbonylation). The high degree of carbonisation along with structural condensation contribute to the increased carbon content during the torrefaction [52]. These findings are in line with the elemental composition analysis of the biomass and align with previous studies reporting similar elemental transitions in thermally pretreated lignocellulosic materials [33,53].

### 3.2. Biosurfactant Production from Torrefied Biomass

After extensive physicochemical characterisation of torrefied biomass at different temperatures, each biomass sample was used as a potential feedstock for biosurfactant production using the non-pathogenic microorganism *B. thailandensis.* As one of the major objectives of this proof-of-concept study was to reduce the overall cost of the process while sustaining microbial growth and biosurfactant production, a modified semi-solid-state fermentation method was used with each torrefied biomass suspended in nutrient broth medium without any added sugars. Conventionally, fermentation of alternative feedstock such as agro-industrial residues is preceded by a hydrolysis step that yields monomeric sugars; we bypassed this step in this study, which has implications for increasing the economic viability of this process [54]. Moreover, the semi-solid-state mode of fermentation ensured that the bacteria had access to nutrient fluxes from the high concentration of torrefied biomass (5% *w*/*v*) used in the medium. A similar rationale has been used in seaweed-based lactic acid bacteria fermentation [55]. Initially, three different concentrations (2.5% (*w*/*v*), 5% (*w*/*v*), and 10% (*w*/*v*)) were studied; however, the most promising results based on emulsification were obtained at 5% (*w*/*v*) biomass loading. At a torrefied biomass concentration of 10% (*w*/*v*), slurry formation and insufficient bacterial growth did not allow for continuation of the experiments.

Untreated lignocellulosic biomass is complex and challenging to fractionate [56]. In our study, the results of fibre analysis suggested that torrefied biomass at lower temperatures (225 °C and 250 °C) contained sufficient structural polymers to sustain the growth of *B. thailandensis.* High cellulose crystallinity was observed in XRD analyses at these temperatures, which has been previously reported to be a deterrent to fermentation. However, we hypothesised that the presence of all the major structural components including hemicellulose, in the beginning of partial decomposition stages in the T225 biomass in particular (as explained in the TGA/DTG results), could support microbial biosurfactant production by *B. thailandensis.* Presumably, the microorganism could attach itself to the ruptures and cracks developed during low-temperature torrefaction, which are not present in raw biomass and not as hydrophobic as those in higher-temperature torrefaction. A previous report by Chen et al. [21] outlined the cellulose degradation ability and denitrification ability of a member of *Burkholderia* spp. in the presence of corn biochar, which suggests the ability of the microorganism to survive and produce extracellular products in the presence of biochar. Moreover, another group has reported the ability of an isolate of *Burkholderia* spp. to produce an enzyme cocktail with lignolytic, hemicellulolytic, and cellulolytic activities [57], which aligned well with our hypothesis, which was confirmed by experimental validation as follows.

#### Preliminary Characterisation of Biosurfactant from Torrefied Biomass

**Figure 7:**
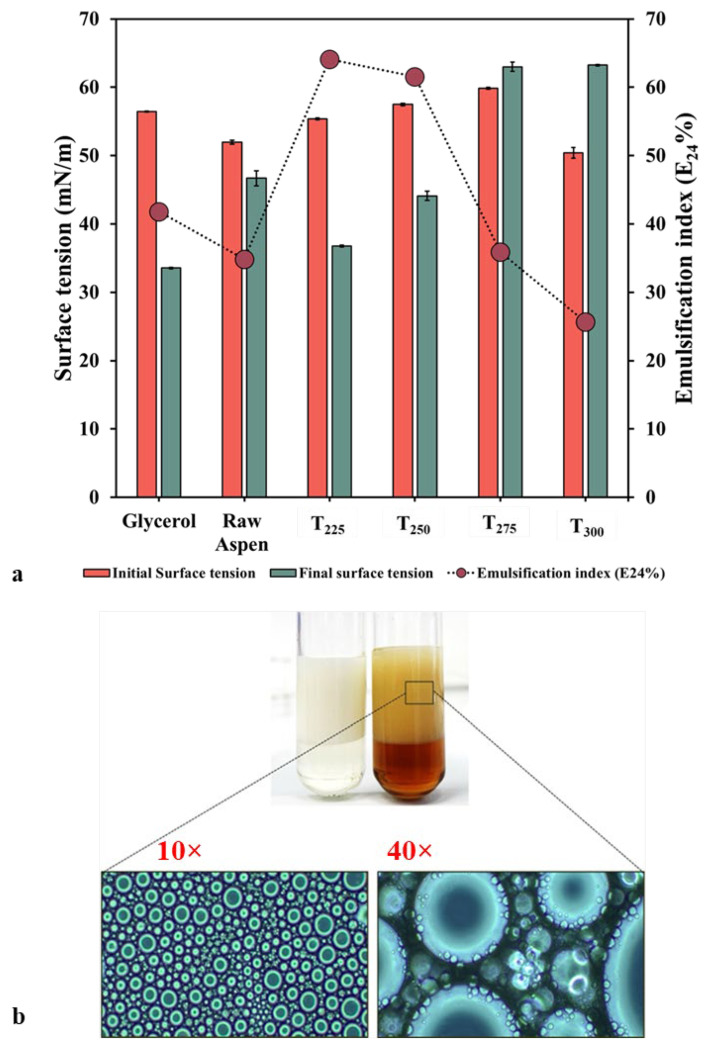
(**a**) Surface tension-lowering ability and emulsification indices of different cell-free supernatants and (**b**) comparison of emulsion stability of 1% (*w*/*v*) CTAB (left) and crude T225 cell-free supernatant (right) with diesel oil (Inset: phase-contrast microscopy images of 40-days-old T225 cell-free supernatant–diesel oil emulsions at 10× and 40× magnification).

The surface tensions of the cell-free supernatants and the respective production media were compared to confirm the production of tensio-active compounds (Figure 7a). It was found that fermentation of biomass torrefied at lower temperatures (225 °C and 250 °C) resulted in the production of tensio-active compounds that lowered the surface tension of the respective production media (from 55.4 to 36.8 mN/m for T225 and from 57.5 to 44.1 mN/m for T250), validating their biosurfactant nature. Predictably, the positive control—production media with glycerol—yielded the best result (a reduction of surface tension by 22.9 units to the final value of 33.6 mN/m). Elshikh et al. [58] reported that *B. thailandensis* grown in the same medium resulted in the surface tension being reduced to 30 mN/m, attributing this to the production of rhamnolipids. In separate studies using the same control production medium, different surface tension values from 30 to 43 mN/m have been reported at different incubation temperatures and times [20,58]. Our results fall into this range, confirming the suitability of our positive control as a comparison for biosurfactant production from alternative feedstocks, particularly since there are no commercial standards available for rhamnolipids produced by *B. thailandensis*. Production media containing raw aspen had initial and final surface tensions of 52 and 46.7 mN/m, respectively, denoting a decrease of 5.3 units only. This clearly demonstrates the beneficial effect of torrefaction at lower temperature in the microbial production of tension-active compounds. However, at higher temperatures (275 °C and 300 °C), the cell-free supernatants had higher surface tension than the respective production media, suggesting that these temperatures are not suitable to produce biosurfactants.

Similar effects were also observed in emulsification assays (Figure 7a). Cell-free supernatants derived from biomass torrefied at lower temperatures (225 °C and 250 °C) could form emulsions with diesel oil (64.1% and 61.5%, respectively) on agitation by vortexing for 2 min. Comparatively, the positive control, 1% (*w*/*v*) CTAB, had an emulsification index of 71.8%. However, in order for the cell-free supernatants of biomass torrefied at higher temperatures (275 °C and 300 °C) to form emulsions (35.9% and 25.6%, respectively), vortexing had to be performed for an extended time (up to 6 min). A stable emulsifier is characterised by its ability to maintain at least 50% of the original emulsion volume 24 h after emulsification [59,60]. Based on this criterion, T275 and T300 did not yield good emulsifiers. Further, these emulsions were not stable beyond 24 h, which is the standard time for measurement. Near 24 h, the initial emulsion layer that formed started slowly disintegrating to a very thin layer. A similar trend was observed for emulsions derived from production media containing glycerol (41.79%) and raw aspen (34.84%). In sharp contrast, the emulsions of T225 cell-free supernatants were found to be stable up to 40 days at room temperature. The height of the T225 emulsion layer stayed constant for the mentioned time period, whereas that of CTAB started reducing (Figure 7b). Phase contrast microscopy images revealed the presence of intact, tightly packed bubbles in the emulsion layer of T225 (Figure 7b inset).

The orcinol method is the most common method used for quantifying biosurfactant production from *B. thailandensis* [20,58]. Although we attempted to quantify the production of biosurfactants in these various production media by the orcinol method, the dark reddish-brown colour of the cell-free supernatants also had high absorption in the wavelength range of 400–500 nm, resulting in very high OD values at 421 nm (Supplementary Figure S1). Therefore, it was not possible to derive an accurate value for any of the production media, except for glycerol (Supplementary Figure S2). Further purification steps based on the structure of the biosurfactant compounds could yield accurate gravimetric measurements of biosurfactant yield in this feedstock. Based on the combined aforementioned results, the authors decided to use the mild torrefaction temperature of 225 °C, which gave more promising results for further biosurfactant production experiments.

**Figure 8:**
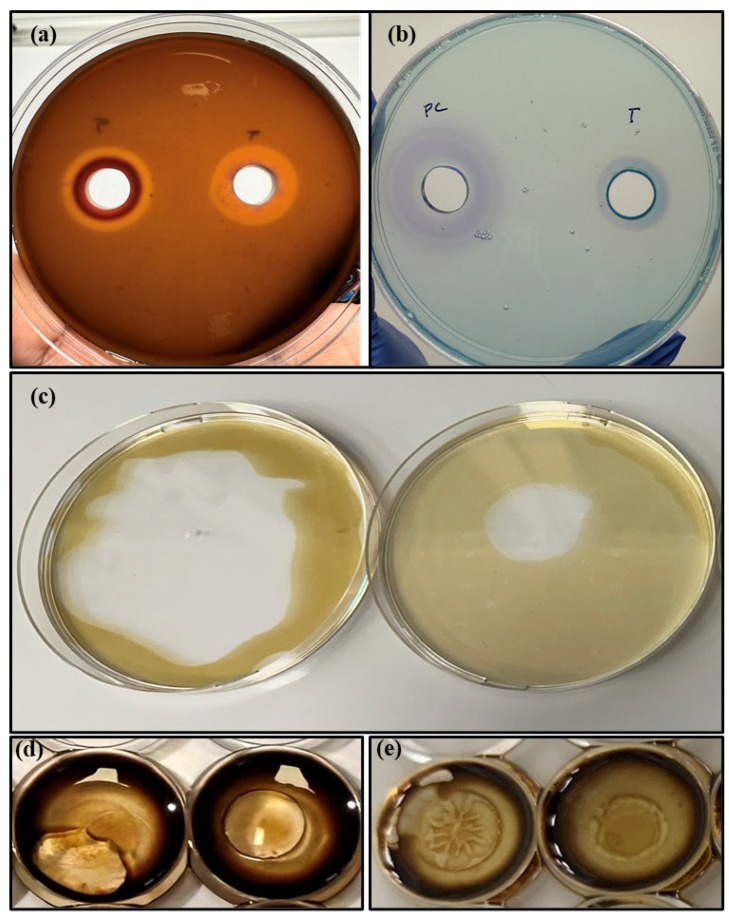
(**a**) Hemolysis assay of T225 biosurfactant (right) versus rhamnolipids grown on glycerol (left); (**b**) CTAB-methylene blue agar assay of T225 biosurfactant (right) versus commercial *Pseudomonas aeruginosa* rhamnolipids (left); and (**c**) oil displacement assay against crude oil of T225 biosurfactant (right) versus rhamnolipids grown on glycerol (left); drop-collapse assay against crude oil of T225 biosurfactant versus rhamnolipids grown on glycerol (**d)** within 2 min and (**e)** after 7 min.

Biosurfactant production in the T225 supernatant was also confirmed using a series of non-quantitative methods, such as the hemolysis assay, CTAB-methylene blue agar assay, and drop-collapse and oil-clearing assay, after adapting the medium constituents for the use of cell-free supernatants instead of live culture [61]. Not all biosurfactants have hemolytic activity [62]; however, the T225 cell-free supernatant exhibited clear β-hemolysis, characterised by a clear zone of approximately 2.2 cm surrounding the well, comparable to the positive control (Figure 8a). In addition, it exhibited a clear blue halo in the CTAB-methylene blue agar assay, which indicated a net negative charge on the crude biosurfactant. The diameter of the halo was less than that formed by the commercial rhamnolipids, suggesting that commercial rhamnolipids have a more net negative charge in their congeners than the T225 cell-free supernatant (Figure 8b).

In the oil displacement activity assay against crude oil (Figure 8c), the T225 cell-free supernatant formed a clear zone; however, its size was much smaller compared to that formed by the rhamnolipids derived from the production media containing glycerol. The concentration of biosurfactant could be low in the T225 cell-free supernatant. A similar concentration-dependent effect was observed in the drop-collapse assay; although the drops of T225 cell-free supernatant collapsed (Figure 8d), it took approximately 5–7 min compared to the instantaneous collapse of the commercial rhamnolipids (Figure 8e). The presence of other compounds in the cell-free supernatant could also have contributed to this effect.

The interfacial tension analyses revealed very good activity of the T225 cell-free supernatant against crude oil and diesel oil, with diesel oil yielding better results. With diesel oil, T225 lowered the interfacial tension to 0.5 mN/m, yielding very similar results as the cell-free supernatant from the production medium with glycerol (0.8 mN/m). With crude oil, T225 had a higher interfacial tension of 4.9 mN/m compared to 1.5 mN/m of the positive control. The formation of much smaller droplets of diesel than crude oil in the denser T225 cell-free supernatants, the lower interfacial tension, and higher emulsification capability together point to the suitability of using T225 cell-free supernatants without purification for low-cost bioremediation of diesel contamination [63].

Herein, we have described a comprehensive characterisation regime of torrefied biomass at different temperatures, with the aim of using it as a substrate for specialty biochemical production, with biosurfactants as an example. The combined characterisation and biosurfactant production results suggest that *B. thailandensis* was able to utilise the structurally and compositionally altered fibre constituents in low-temperature torrefied biomass (T225 and T250) as feedstocks for biosurfactant production.

## 4. Conclusions and Future Perspectives

An integrated strategy of low-temperature torrefaction of waste wood chips that are readily available in Estonia, followed by direct microbial conversion, has been outlined for a proof-of-concept of biosurfactant production. While no techno–economic analyses were conducted in this study, three points are to be considered: (1) waste wood chips that would otherwise be used for district heating have been repurposed for a high-demand specialty chemical, which can be (2) processed in existing torrefaction plants across Europe, in milder conditions, and (3) the high surface tension- and interfacial tension-lowering as well as emulsification of the T225 biosurfactant suggest its direct utilisation without intensive purification in applications that require low-cost biosurfactants (such as oil bioremediation). However, the development of a biosurfactant purification scheme based on the structure of the biosurfactant compounds followed by yield optimisation could aid in further process improvement, particularly in specialty applications such as food emulsifiers.

A key consideration for the commercial application of this process is scaling up of the semi-solid-state fermentation process with optimal process control. For this, the most appropriate reactor configurations, such as static bed reactors with mixing capabilities or pneumatic reactors, could be tested, along with culture optimisation. However, challenges in downstream processing exist to date in such configurations. The concept of multiple reuses of solid substrates such as wood waste could be tested and adapted to commonly used reactor configurations, to alleviate some of these concerns. To the best of our knowledge, this work is the first report of the direct utilisation of torrefied biomass for biosurfactant production. This work represents an integrated chemical-free pretreatment and biosurfactant production strategy that combines a cheap, abundantly available substrate and focused microbe-mediated fermentation with the potential to improve process efficiency.

## Figures and Tables

**Table 1:** Ultimate analysis of raw and torrefied aspen biomass.

Sample	C (%)	H (%)	N (%)	O (%)	S (%)	Atomic Ratio	
						H/C	O/C
Raw aspen	47.7 ± 0.1	7.0 ± 0.0	0.2 ± 0.0	45.0 ± 0.1	0.04	0.15	0.94
T225	49.4 ± 0.2	7.1 ± 0.2	0.6 ± 0.7	42.9 ± 0.8	0.02	0.14	0.87
T250	49.6 ± 0.3	6.7 ± 0.1	0.1 ± 0.0	43.6 ± 0.3	0.01	0.14	0.88
T275	55.6 ± 0.1	6.3 ± 0.1	0.1 ± 0.1	38 ± 0.0	0.01	0.11	0.68
T300	61.4 ± 0.5	5.9 ± 0.1	0.1 ± 0.0	32.6 ± 0.5	0.01	0.10	0.53

## Data Availability

The original contributions presented in this study are included in the article/Supplementary Materials. Further inquiries can be directed to the corresponding author.

## Supplementary Materials

The following supporting information can be downloaded at https://www.mdpi.com/article/10.3390/polym17131808/s1, Supplementary Figure S1: Absorbance of nutrient media containing T225; Supplementary Figure S2: Biosurfactant concentration in media with different types and amount of torrefied wood waste.
