# Supplementary material for: Repurposing Torrefied Biomass as a Novel Feedstock for Microbial Bioprocessing—A Proof-of-Concept of Low-Cost Biosurfactant Production
Source: Polymers (Basel). 2025 Jun 29;17(13):1808. doi: 10.3390/polym17131808 (PMC12251772; doi:10.3390/polym17131808)

Supplementary Information

Repurposing torrefied biomass as a novel feedstock for microbial biorefineries – A proof-of-concept of low-cost biosurfactant production

Anjana Hari[1], Vahur Rooni[1], Udayakumar Veerabagu[1], Shiplu Sarker[2], Alar Konist[3], Timo Kikas[1]

[1]*Biosystems Engineering, Institute of Forestry and Engineering, Estonian University of Life Sciences, Kreutzwaldi 56, Tartu 51014, Estonia*

[2] *Department of Manufacturing and Civil Engineering, Faculty of Engineering, Norwegian University of Science and Technology, 2815 Gjøvik, Norway*

[3]*Department of Energy Technology, Tallinn University of Technology, Tallinn 19086, Estonia*

Corresponding author

Prof. Timo Kikas

Email: timo.kikas@emu.ee

Supplementary figure S1

Absorbance of nutrient media containing T225

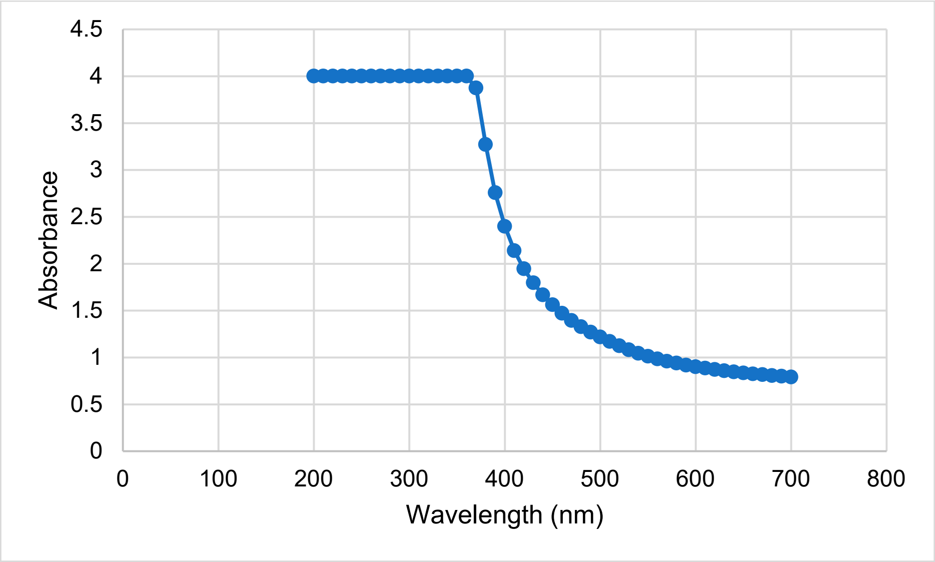

Supplementary figure S2

Biosurfactant concentration in media with different types and amount of torrefied wood waste

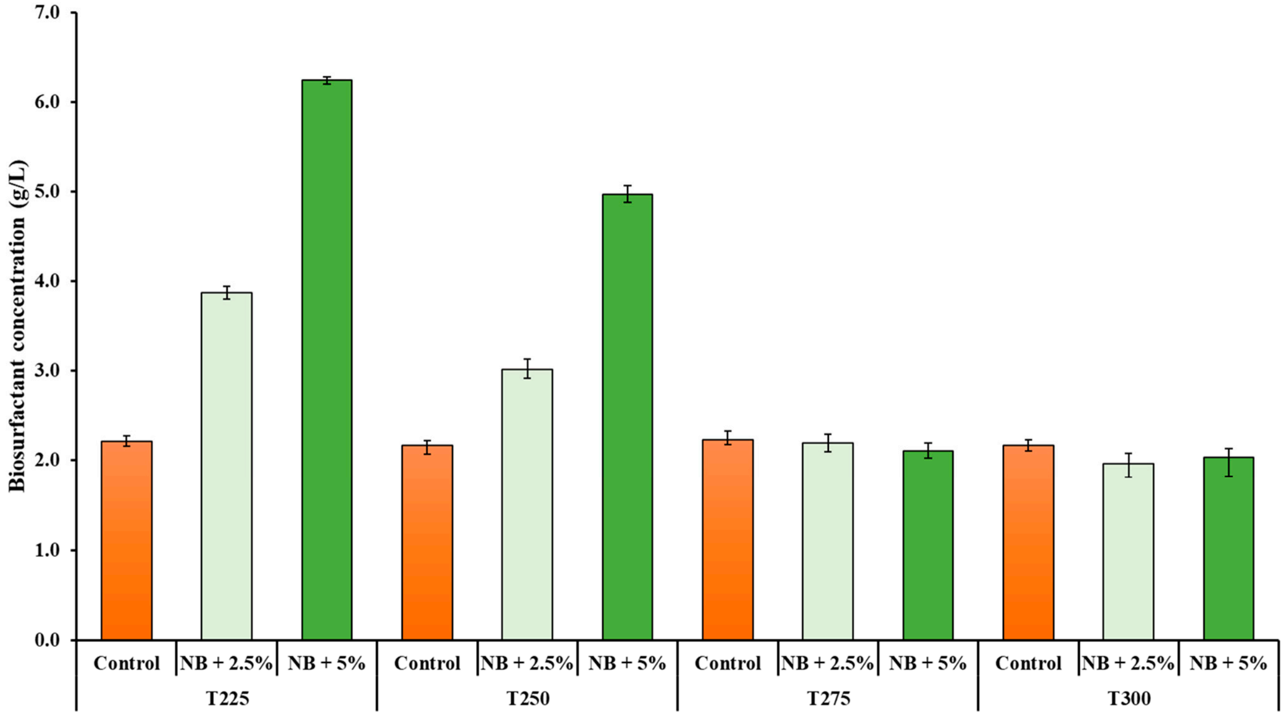

Supplement: Supplementary file 1 [file polymers-17-01808-s001.zip › polymers-3714122-supplementary.pdf]
